# Rapunzel Syndrome in a Pediatric Female Patient: A Case Report of Trichobezoar Causing Intestinal Obstruction

**DOI:** 10.7759/cureus.101899

**Published:** 2026-01-20

**Authors:** Ali T Alamer, Osama Kattih, Ahmed A Al-Amoudi, Husain A Althani, Abdulaziz J Almarzooq

**Affiliations:** 1 Pediatrics, Almoosa Specialist Hospital, Al-Ahsa, SAU; 2 Pediatric Intensive Care Unit (PICU), Almoosa Specialist Hospital, Al-Ahsa, SAU

**Keywords:** gastrointestinal bezoar, gastrointestinal obstruction, inpatient pediatrics, pediatric intensive care unit(picu), rapunzel syndrome

## Abstract

Rapunzel syndrome is a very rare type of trichobezoar in which a hair mass extends to the stomach and small intestine forming a body and tail. It usually affects young females with a psychiatric background. Symptoms are usually nonspecific and often delay the diagnosis.

We present in this report a case of a six-year-old previously healthy girl who presented with two months of intermittent abdominal pain, vomiting, and constipation. Initial laboratory tests and two times abdominal ultrasounds were unremarkable. Abdominal X-ray showed moderate dilatation of the bowel. Further physical examination revealed patchy scalp alopecia, raising suspicion for hair ingestion. Upper endoscopy showed a large trichobezoar in the stomach and duodenum, but endoscopic removal failed. The patient underwent open laparotomy, and an 11 × 4 cm bezoar with a tail extending into the duodenum was removed.

This case highlights the value of recognizing Rapunzel syndrome in children with vague gastrointestinal symptoms, even without a prior psychiatric history. Early recognition and management, including surgical and non-surgical interventions, are important to prevent complications and recurrence.

The study aims to describe a rare presentation of Rapunzel syndrome in order to increase awareness among physicians and emphasize the importance of early recognition and treatment to prevent serious complications.

## Introduction

Bezoar is derived from the Persian word “Padzahr,” which means antidotes, as they believed historically that some materials from plants or animals have anti-poison effects. However, in recent medical terminology, bezoar means an accumulation of ingested materials that are insoluble or indigestible in the gastrointestinal tract [1-7].

Rapunzel syndrome is an extremely rare and unusual form of gastric trichobezoar, characterized by the accumulation of hair in the stomach, which may extend into the small intestine and reach the ileum, forming a Bezoar with a tail [1]. It usually affects children and adolescents, specifically females with a history of psychiatric disorders such as hair-pulling (trichotillomania) and hair-eating (trichophagia), or children with trisomy 21 [2]. The clinical presentation of Rapunzel syndrome is usually vague gastrointestinal symptoms such as generalized abdominal pain, nausea, vomiting, weight loss, or the presence of a palpable abdominal mass [3]. Most of the time, diagnosis is delayed or missed, and many patients are diagnosed when there is a clinical suspicion or when the trichobezoar becomes large enough to cause gastric or intestinal obstruction [4]. Complications of untreated Rapunzel syndrome include gastric or small bowel obstruction, gastric ulcers, perforation, gastrointestinal bleeding, and anemia [5]. Early recognition and interventions, including behavioral therapy, are crucial to avoid complications and surgical intervention [6].

Here, we report a case of Rapunzel syndrome in a six-year-old female patient previously healthy, who presented with vague abdominal pain and vomiting, with no previous psychiatric history, emphasizing the importance of clinical suspicion even when psychiatric history is absent. We talk over diagnostic challenges, surgical and non-surgical management, including dietary and psychiatric follow-up.

This study aims to describe a rare presentation of Rapunzel syndrome in order to increase awareness among physicians and emphasize the importance of early recognition and treatment to prevent serious complications.

## Case presentation

A six-year-old previously healthy female patient presented to the pediatrics ER with a history of recurrent generalized abdominal pain for around two months, associated with vomiting containing gastric contents and constipation for three weeks. Two previous abdominal ultrasounds were done and were unremarkable.

On examination, the patient was conscious, alert, active, not jaundiced or pale, with no signs of dehydration or respiratory distress. Weight and height were within the 50th percentile. Abdominal examination was unremarkable with no palpable mass. Initial laboratory tests are shown in Table 1.

**Table 1:** Initial laboratory results All lab results were within normal range. WBC: White blood cell; AST: Aspartate transaminase; ALT: Alanine transaminase; TIBC: Total iron-binding capacity

Test	Result	Reference value
WBC	6.5x10^3^/uL	5-15.5x10^3^/uL
Hemoglobin	12.8 g/dL	11.5-15.5 g/dL
Platelets	250x10^3^/uL	150-350x10^3^/uL
AST	30 U/L	21-44 U/L
ALT	22 U/L	9-25 U/L
Na	139 mEq/L	135-145 mEq/L
K	4.2 mEq/L	3.5-5 mEq/L
Ferrtin	18 Ng/mL	14-79 Ng/mL
TIBC	380 mcg/dL	250-400 mcg/dL
Iron	45 mcg/dL	16-128 mcg/dL
Glucose	88 mg/dL	70-140 mg/dL

Stool culture for bacterial pathogens was negative, and stool was negative for viral agents, ova, and parasites. Fecal blood occult was negative.

**Figure 1:**
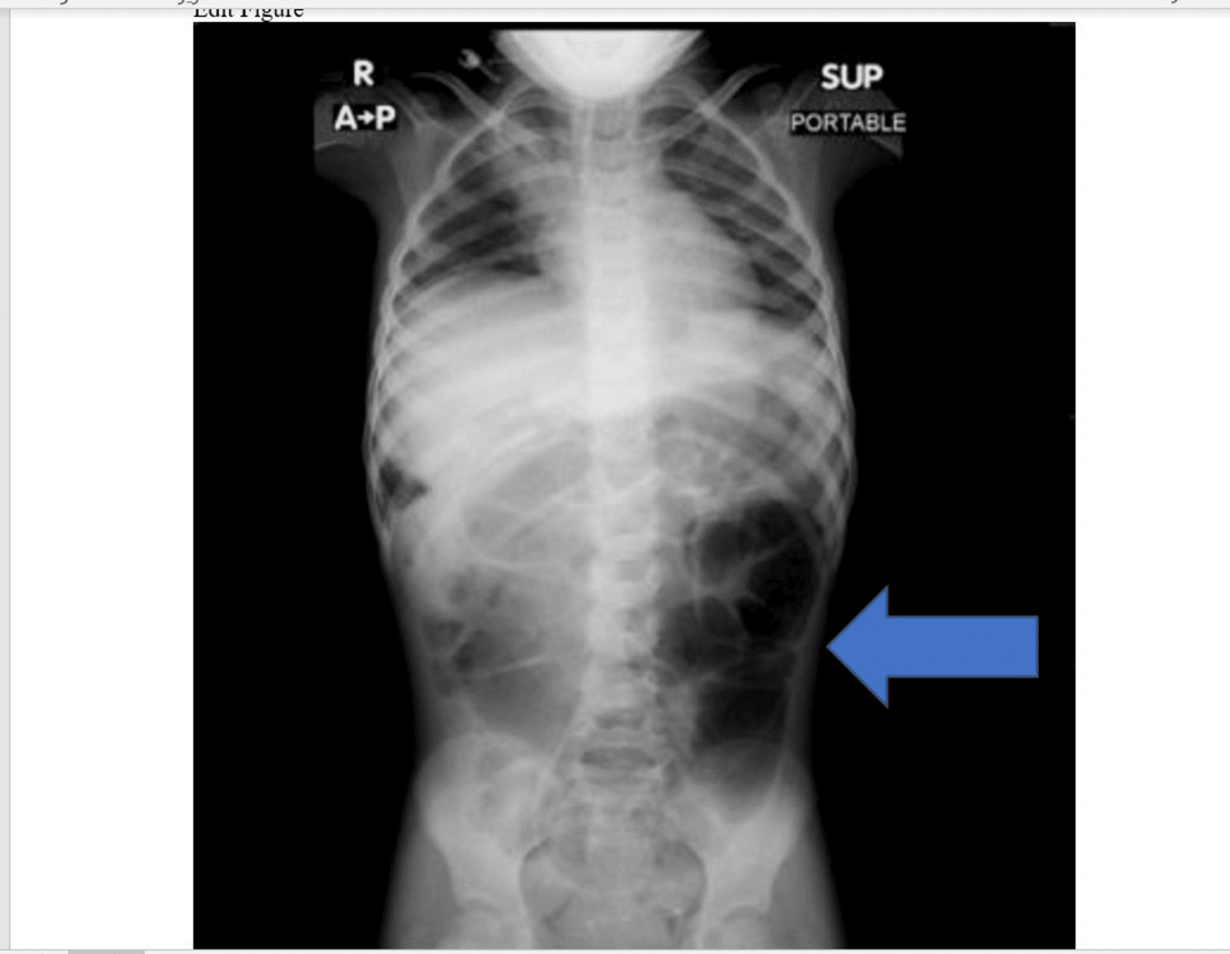
Abdominal X-ray shows moderate dilatation of bowels Antero-posterior erect abdominal X-ray showing moderate dilatation of bowels as shown by the arrow.

Upon PICU admission, an abdominal X-ray was done and showed moderate dilation of the bowels (Figure 1). Abdominal ultrasound was repeated as normal.

**Figure 2:**
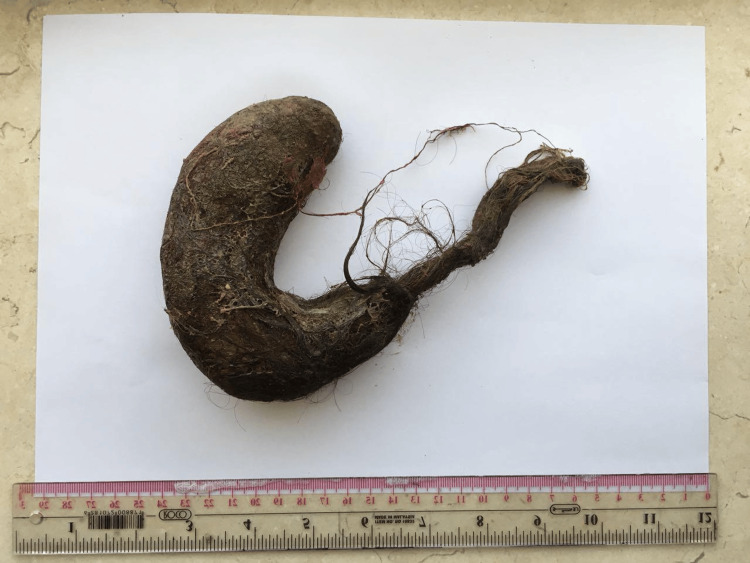
11 cm x 4 cm trichobezoar specimen showing tail.

**Figure 3:**
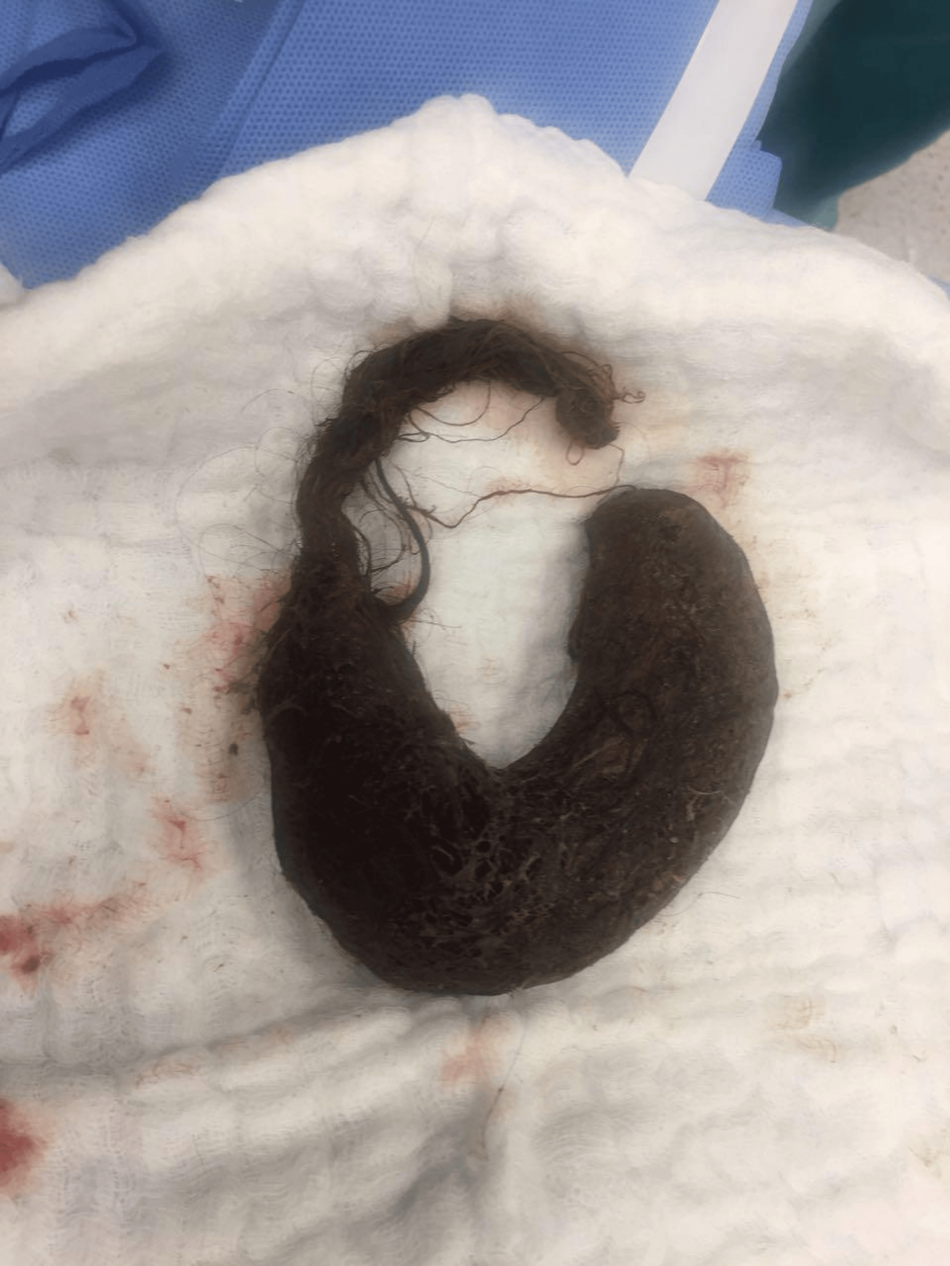
11 cm x 4 cm trichobezoar specimen showing tail.

On detailed physical examination, patches of alopecia were seen on the head, which raised the suspicion of foreign body ingestion. As per the recommendations of the gastrointestinal consultant, the patient underwent upper gastrointestinal endoscopy. A mass of hair was found in the stomach and duodenum, but could not be removed by endoscopy. Upon surgical consultation while the patient was in the operation room, laparotomy was recommended to remove the hair mass. After open laparotomy, a mass of hair was removed from the stomach and duodenum, measuring around 11 cm x 4 cm bezoar specimen showing tail (Figures 2-3).

One day later, the patient was discharged home with a psychiatry appointment for further management to prevent recurrence.

## Discussion

Ingestion of hair leads to the formation of a trichobezoar in the stomach because hair is indigestible and resistant to bowel peristalsis. Over time, it accumulates and forms a solid mass; when the mass extends beyond the stomach into the small bowel, the condition is termed Rapunzel syndrome [1,2]. Although bezoars of different kinds are common, trichobezoars represent a minority of all human bezoars. Rapunzel syndrome is extremely rare, with less than 100 cases reported and published worldwide [1,3]. Rapunzel syndrome usually presents with non-specific GI symptoms, generalized abdominal pain, vomiting, weight loss, signs of obstruction such as constipation or a palpable abdominal mass, and sometimes nutritional deficiencies [3-6]. Because symptoms are vague and often chronic, suspicion may be low; thus, imaging (ultrasound, CT) and/or endoscopy are needed to establish a diagnosis [3-6]. Endoscopic removal may be enough for small trichobezoars; however, in numerous cases, especially in those with large bezoars expanding into the small bowel, surgical intervention such as laparotomy is needed [3-6]. After management, nutritional support and psychiatric counselling are critical to prevent recurrence [5,6].

## Conclusions

Even though Rapunzel syndrome is extremely rare, it should be considered as a differential diagnosis of young female patients, specifically if they have a psychiatric background presenting with chronic nonspecific gastrointestinal symptoms, weight loss, or a palpable abdominal mass, even in the absence of a clear history of hair-pulling or witnessed hair-eating by family. Early confirmation of diagnosis using imaging and endoscopy, followed by rapid surgical removal with good psychiatric intervention, is crucial to reduce life-threatening complications and prevent recurrence. Reporting of such cases remains important to boost awareness among physicians and improve early management.

## References

[1] Ahmed Bashir E, Samiullah Samiullah, Attique Sadiq M, Yusuf O, Khan K (2010). Rapunzel syndrome. J Ayub Med Coll Abbottabad.

[2] Aziz Khan Y, Zaib Z, Tufail F, Rafiq K, Muhammad K (2024). Every tangle has a story - Rapunzel syndrome: a case report. J Pediatr Adolesc Surg.

[3] Al-Wadan AH, Al-Absi M, Al-Saadi AS, Abdoulgafour M (2006). Rapunzel syndrome. Saudi Med J.

[4] Modh FA (2018). Rapunzel syndrome: a review of unusual case. Int Surg J.

[5] Vora S, Godhani C, Patel S (2019). Rapunzel syndrome in a 7-year-old boy: a case report. Int J Surg Sci.

[6] Cannalire G, Conti L, Celoni M (2018). Rapunzel syndrome: an infrequent cause of severe iron deficiency anemia and abdominal pain presenting to the pediatric emergency department. BMC Pediatr.

[7] Phillips MR, Zaheer S, Drugas GT (1998). Gastric trichobezoar: case report and literature review. Mayo Clin Proc.

